# Sequelae of Anticoagulant Therapy in a Patient with History of Pulmonary Malignancy: A Case Report

**DOI:** 10.5811/cpcem.2020.7.48317

**Published:** 2020-10-05

**Authors:** Christopher B. Gilsdorf, Hillary E. Davis

**Affiliations:** * University of Tennessee Medical Center, Department of Emergency Medicine, Knoxville, Tennessee; † University of Tennessee Medical Center, Department of Family Medicine, Knoxville, Tennessee

**Keywords:** anticoagulation, malignancy, intracranial hemorrhage, venous thromboembolism, brain metastases

## Abstract

**Introduction:**

In patients with known malignancy and possible intracranial metastatic disease who are receiving treatment with therapeutic anticoagulation, limited data exist regarding risk of intracranial bleeding.

**Case Report:**

We present a case of a 64-year-old female with known lung malignancy, evidence of possible metastatic disease, and bilateral deep vein thrombosis, who suffered severe intracranial hemorrhage following initiation of therapeutic anticoagulation. Current guidelines, available risk- stratification tools, and treatment options with their risks are discussed.

**Conclusion:**

In patients with known or suspected intracranial metastatic disease, clinical decision tools can assist both the clinician and the patient in weighing risks and benefits of anticoagulation.

## INTRODUCTION

Therapeutic anticoagulation is used in a variety of clinical settings to prevent the feared sequelae of venous thromboembolism (VTE), including thrombus propagation, venous insufficiency, and pulmonary embolus (PE), in patients with predisposing conditions including hypercoagulable states, atrial fibrillation, and mechanical cardiac valve replacements. However, initiation of anticoagulation is not without significant risk for hemorrhage, particularly in certain patient populations. Several evidence-based clinical decision tools aid in risk stratification of at-risk patients but do not explicitly address known malignancy.^1^ We present a case in which anticoagulation therapy was initiated in a patient with a pre-existing neoplasm with possible metastatic spread that resulted in an unfortunate patient outcome likely secondary to undiagnosed intracranial metastases.

## CASE REPORT

A 64-year-old White female with history of stage I squamous cell carcinoma of the right middle lung, renal transplant secondary to membranous glomerulonephritis, history of previous VTE, hypertension, chronic obstructive pulmonary disease, and stage four chronic kidney disease presented to the emergency department (ED) for treatment of deep venous thrombosis (DVT). The patient had been sent by her pulmonologist after obtaining outpatient, lower-extremity venous Doppler ultrasounds earlier that day. The patient had been recently hospitalized for an episode of pneumonia and discharged two weeks prior during which her warfarin had been discontinued for unclear reasons; her history of stage I (T1a, N0) squamous cell cancer of the lung had been only minimally addressed during this admission. The patient had not had a positron emission tomography to assess her tumor staging in nearly 10 months. Additionally, her most recent oncology notes from six months prior following two treatments of stereotactic ablative radiotherapy demonstrated a stable computed tomography (CT) of the thorax and recommended surveillance CT in six months. However, a CT of the abdomen obtained three weeks prior to her ED visit to assess for urinary pathology showed a nonspecific 1.9-centimeter (cm) hypodensity of the liver, potentially concerning for metastatic disease.

On the day of her ED evaluation, she endorsed right lower leg swelling without redness and right leg pain causing difficulty with ambulation. She denied weakness or sensory loss, bladder or bowel dysfunction, headache, fever, chest pain, dyspnea, and all other review of systems. The patient’s presenting vital signs were grossly normal as she was afebrile (36.2° Celsius) with a heart rate of 81 beats per minute, respiratory rate of 16 breaths per minute, blood pressure of 136/82 millimeters of mercury (mm Hg), and an oxygen saturation of 97% on room air. The patient’s physical examination was remarkable for mild tenderness in the posterior aspect of the right upper, middle, and lower leg, with intact distal neurovascular status. There was no overlying erythema or edema. The rest of her physical examination was grossly normal, including a neurologic examination without any deficits.

The patient’s laboratory workup was remarkable for a creatinine of 2.07 milligrams per deciliter (mg/dL) (normal range 0.57–1.00 mg/dL) and estimated glomerular filtration rate of 25 (normal >58), elevated leukocyte count of 13.2 thousand (K)/microliter (μL) (normal range 3.4–10.8 K/ μL), platelet count of 96 K/μL (normal range 150–379), prothrombin time of 13 seconds (normal range 9.1–12.0), international normalized ratio (INR) of 1.26 (normal range 0.80–1.20), and partial thromboplastin time of 27.6 seconds (normal range 24.4–31.4). Her lower-extremity venous Doppler studies, reviewed upon arrival in the ED, demonstrated acute deep venous occlusive disease of the bilateral peroneal veins and the right common femoral vein in addition to acute superficial occlusion of the right greater saphenous vein.

Given the patient’s prior history of VTE, previous renal transplant, and current findings of bilateral DVT, both the vascular surgery and transplant services were consulted; both recommended initiation of intravenous heparin infusion for full anticoagulation treatment. Heparin bolus and drip were initiated. The hospitalist was consulted to admit the patient, agreed with the plan for therapeutic heparin infusion, and noted the patient would now require lifelong anticoagulation given that this was her second episode of VTE. The hematology/oncology service was consulted, but did not evaluate the patient the day of admission. The patient had a non-contrast CT of the thorax performed shortly after initiation of heparin to evaluate for persistent pneumonia. This study demonstrated an enlarging hepatic lesion consistent with metastatic disease that had increased in diameter from 1.9 cm to 2.4 cm over the prior three weeks.

CPC-EM Capsule

What do we already know about this clinical entity?

*Venous thromboembolic disease is a common comorbid condition in patients suffering from malignancy, and anticoagulant pharmacotherapy is considered standard of care*.

What makes this presentation of disease reportable?

*We discuss a patient with known malignancy initiated on anticoagulation despite possible metastatic disease and who suffered a poor outcome*.

What is the major learning point?

*In treating venous thromboembolism secondary to malignancy, risk-stratification tools, type of malignancy, and shared decision-making should guide treatment*.

How might this improve emergency medicine practice?

*Knowledge of indications and contraindications for acute anticoagulant use in the setting of malignancy can guide proper pharmacotherapy and avoid adverse events*.

**Image 1:**
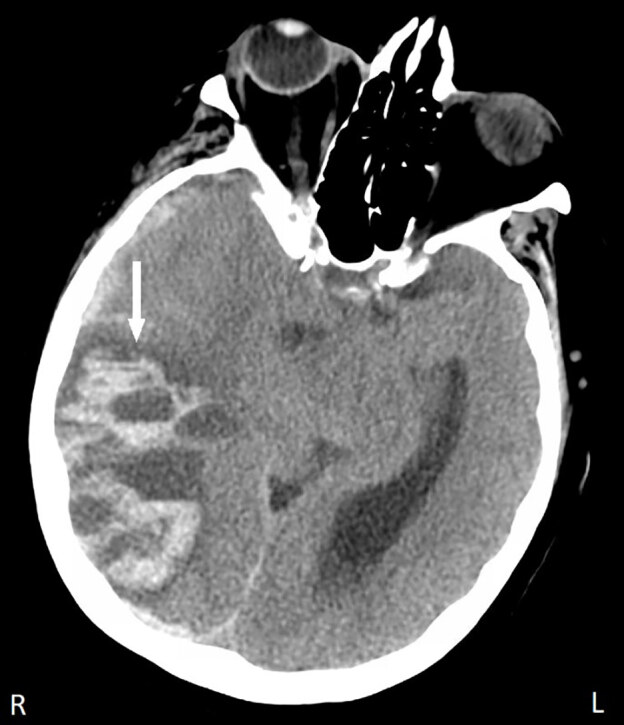
Axial view of noncontrast computed tomography of the brain depicting a large right-sided parietal/temporal/occipital hemorrhage (arrow) with right-to-left midline shift in a 64-year-old female with history of non-small-cell lung cancer following initiation of therapeutic heparin anticoagulation for treatment of deep venous thrombosis.

**Image 2:**
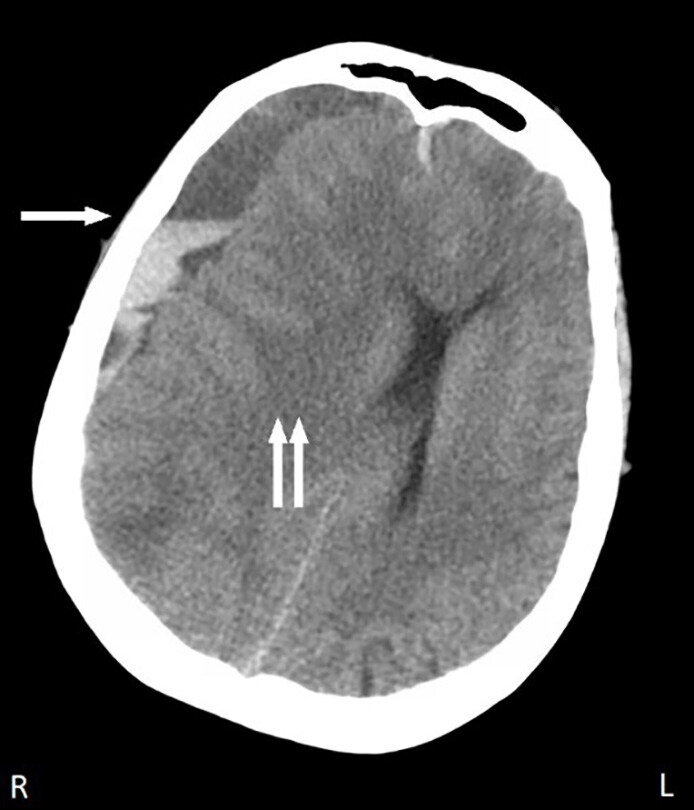
Axial view of noncontrast computed tomography of the brain depicting a right-sided acute-on-chronic subdural hematoma (arrow) with right-to-left midline shift and ventricular obliteration (double arrow) in a 64-year-old female with history of non-small-cell lung cancer following initiation of therapeutic heparin anticoagulation for treatment of deep venous thrombosis.

Six hours after admission, the patient developed a headache. Two hours later she subsequently developed lethargy and confusion, which progressed over minutes to obtundation. The patient was tachypneic, possessed anisocoria, and was hypertensive to a systolic blood pressure of 200 mm Hg. The hospitalist discontinued the heparin drip, called for a code intubation, ordered protamine, and transferred the patient to the intensive care unit. A non-contrast CT head was performed to evaluate for suspected intracranial hemorrhage (ICH). Her CT demonstrated a large right parietal/temporal/occipital hemorrhage and a right subdural hematoma accompanied by 1.8 cm right-to-left midline shift, uncal herniation, and contralateral brainstem compression (Images 1 and 2). The radiologist did not address potential metastatic etiology of her bleed.

The neurosurgery service was consulted and a craniotomy was offered to the patient’s family but was declined after being counseled on the patient’s likely “poor prognosis” even after intervention. Instead, the patient’s family opted to pursue comfort measures. The patient was terminally extubated later that day and shortly thereafter died.

## DISCUSSION

VTE is a significant cause of morbidity and mortality in patients with malignancy; it is in fact the leading cause of death in patients suffering malignancy after cancer itself.^2^ Therapeutic anticoagulation has proven to reduce morbidity and mortality in this population.^3^ While the patient experienced a poor outcome following initiation of heparin in this case, she had been on warfarin within two weeks of her ED visit. Thus, it was surprising that the patient experienced an intracranial bleed. We hypothesize that this consequence was a result of progression and spread of her underlying malignancy. As patients without fully characterized malignancies present with increasing frequency to the ED, it is important for healthcare providers to familiarize themselves with the indications, benefits, risks and alternatives of anticoagulation therapies for the treatment of VTE.

While warfarin, unfractionated heparin (UFH) and low-molecular-weight heparin (LMWH), fondaparinux, and direct oral anticoagulants (DOAC) are all commonly used in the acute treatment of VTE in patients with malignancy, evidence demonstrates a slight mortality benefit in LMWH over UFH in this patient population; its once-daily dosing also makes it an attractive option over UFH.^4^,^5^ Therefore, LMWH is considered first-line therapy for immediate anticoagulation after diagnosis in the first five days of therapy, until concurrently initiated warfarin therapy has reached therapeutic levels.^4^,^5^ This patient’s poor renal function and potential interaction with her immunosuppressant medications unfortunately precluded LMWH use. DOACs are used in some patients with malignancy, but barriers to their use exist. Due to shared metabolic pathways with DOACs, some chemotherapeutic agents may be less efficacious, with concurrent increased risk of bleeding; additionally, vomiting reduces gastrointestinal absorption of DOACs.^5^

Pre-existing evidence of metastatic disease had never been definitively confirmed in this patient. However, the enlarging hepatic nodule noted on multiple CTs was concerning for interim development of disease spread. Given her outcome, it must be considered whether initiation of anticoagulation was inappropriate in her clinical setting. Based on the available evidence, the answer is not entirely clear, but the patient lacked absolute contraindications and had only limited relative contraindications to anticoagulant therapy.^6^ One example was the patient’s thrombocytopenia as her platelet count was 96,000; this put her at increased risk of ICH, but she was not in the highest risk group of severe thrombocytopenia, defined in one study as a platelet count less than 50,000.^7^ Elevated prothrombin time was in fact found to be more predictive of risk of ICH.^8^

Several risk-stratification tools exist to attempt to quantify a given patient’s risk of major hemorrhagic event on anticoagulation, which can serve as an aid to shared decision-making between physician and patient. The HAS-BLED (**H**ypertension, **A**bnormal liver/renal function, **S**troke history, **B**leeding history or predisposition, **L**abile INR, **E**lderly, **D**rug/alcohol usage) scoring system, validated for use in patients receiving anticoagulation for VTE prevention in atrial fibrillation, is frequently used for risk stratification and risk assessment, but does not list current or previous malignancy among its scoring criteria.^1^ The ATRIA (anticoagulation and risk factors in atrial fibrillation) study is another risk-stratification tool for bleeding, created for patients on warfarin; it likewise does not address malignancy as a scoring criterion.^9^ A third, comprehensive tool, HEMORR_2_HAGES (**H**epatic or renal disease, **E**thanol abuse, **M**alignancy, **O**lder than 75 years, **R**educed platelet count or function, **Hypertension**, **A**nemia, **G**enetic factors, **E**xcessive fall risk, **S**troke), does take into account pre-existing malignancy.^10^ However, the HEMORR_2_HAGES tool, essentially a composite of multiple other risk-stratification scoring systems, does not clearly define cutoffs or criteria for several of its components, including hepatic or renal disease.

The type of intracranial lesion matters when it comes to risk of ICH on therapeutic anticoagulation; gliomas were found in one meta-analysis to confer higher risk compared to many other primary or secondary brain lesions. The risk of spontaneous ICH was fourfold higher in metastatic renal cell carcinoma and melanoma than in non-small cell lung cancer (NSCLC) and breast cancer, although independent of anticoagulant use.^11^,^12^ Patients with brain metastases from NSCLC had a relatively low risk of spontaneous bleeding on anticoagulation, with a study showing a rate of only 1.2% over 580 person-years. However, this result may be biased as all patients in these studies received directed radiotherapy, which has been proven to blunt angiogenesis.^13^,^14^ The incidence of spontaneous bleeding in patients not receiving radiotherapy is unknown. Therefore, due to this patient’s primary tumor, she would not have been considered high risk for ICH from anticoagulation therapy.

In this patient, no advanced brain imaging was obtained prior to initiating therapeutic anticoagulation. There is a lack of consensus statements in the literature addressing the need for advanced imaging in similar patient groups to rule out brain metastases prior to initiating anticoagulation.^15^ One approach is to examine both the risk of brain metastases and the likelihood of such metastases to bleed, if present.^15^ Patients with high-risk neoplasms, headaches, or focal neurological deficits should undergo imaging, preferably with magnetic resonance imaging, if therapeutic anticoagulation could be safely delayed.^15^ Otherwise, alternative treatment modalities such as inferior vena cava filters or initiating anticoagulation with available reversal agents may need to be considered.

## CONCLUSION

We presented a case of intracranial hemorrhage following re-initiation of anticoagulation therapy for acute venous thromboembolism in a patient with known pre-existing lung neoplasm with findings concerning for metastatic progression. In select patients with known or suspected intracranial metastatic disease, the risks and benefits of initiation of anticoagulation need to be carefully weighed by both the clinician and patient.
